# A factorial randomized controlled trial to evaluate the effect of micronutrients supplementation and regular aerobic exercise on maternal endothelium-dependent vasodilatation and oxidative stress of the newborn

**DOI:** 10.1186/1745-6215-12-60

**Published:** 2011-02-28

**Authors:** Robinson Ramírez-Vélez, Miryam Romero, Isabella Echeverri, José Guillermo Ortega, Mildrey Mosquera, Blanca Salazar, Sandra Lorena Girón, Wilmar Saldarriaga, Ana Cecilia Aguilar de Plata, Julio Cesar Mateus

**Affiliations:** 1 Escuela de Ciencias Básicas, Universidad del Valle. Calle 4B 36-00. San Fernando, Cali, Colombia; 2 División Salud, Fundación FES Social. Avenida 8 Norte 22 AN -15. Cali, Colombia; 3 Escuela de Salud Pública, Universidad del Valle Calle 4B 36-00. San Fernando, Cali, Colombia

## Abstract

**Background:**

Many studies have suggested a relationship between metabolic abnormalities and impaired fetal growth with the development of non-transmissible chronic diseases in the adulthood. Moreover, it has been proposed that maternal factors such as endothelial function and oxidative stress are key mechanisms of both fetal metabolic alterations and subsequent development of non-transmissible chronic diseases. The objective of this project is to evaluate the effect of micronutrient supplementation and regular aerobic exercise on endothelium-dependent vasodilation maternal and stress oxidative of the newborn.

**Methods and design:**

320 pregnant women attending to usual prenatal care in Cali, Colombia will be included in a factorial randomized controlled trial. Women will be assigned to the following intervention groups: *1. Control group: *usual prenatal care (PC) and placebo (maltodextrine). *2. Exercise group: *PC, placebo and aerobic physical exercise. *3. Micronutrients group: *PC and a micronutrients capsule consisting of zinc (30 mg), selenium (70 μg), vitamin A (400 μg), alphatocopherol (30 mg), vitamin C (200 mg), and niacin (100 mg)*. 4. Combined interventions Group: *PC, supplementation of micronutrients, and aerobic physical exercise. Anthropometric measures will be taken at the start and at the end of the interventions.

**Discussion:**

Since in previous studies has been showed that the maternal endothelial function and oxidative stress are related to oxidative stress of the newborn, this study proposes that complementation with micronutrients during pregnancy and/or regular physical exercise can be an early and innovative alternative to strengthen the prevention of chronic diseases in the population.

**Trial registration:**

NCT00872365.

## Background

In the last decades, it has been suggested that maternal factors such as endothelial function and oxidative stress are key mechanisms in fetal metabolic alterations and the subsequent development of non-transmissible chronic diseases [NTCD] in the adulthood [1].

In addition to the effect of these factors, other external factors such as lifestyle, physical activity, diet, and tobacco consumption could be involved in the development and progress of non-transmissible chronic diseases [1] However, the interaction between the maternal and the external factors during pregnancy that could be involved in the development and progression of NTCD remains unclear. Therefore, it is necessary to establish if maternal and external factors have influence on the placental function and on the neonatal metabolic alterations or both.

During pregnancy, several factors could affect the organization of fetal systems and sub-systems [2-4]. It has been proposed that interventions such as the supplementation with micronutrients and the practice of physical exercise could decrease the incidence or progression of NTCD, probably because both interventions have been reported to increase the synthesis of nitric oxide, a very important factor for the function of the vascular endothelium and for reduction of the oxidative stress [3-5].

This postulate has been primarily based on evidence that ingestion of some micronutrients (*e.g*, vitamin and minerals, among others, etc.) [6] and regular physical activity (at least of 12 weeks) [7], decreases oxidative stress [8] and restore endothelial function [9] in non-pregnant women [10]. However, the effect of these interventions have not been studied during pregnancy, although both are safe and economically feasible [11]. Furthermore, most studies have focused on measuring the effects on anthropometric measurements of neonate (*i.e.*, weight, height, among others) [12-14] despite the possible independent relationship between the endogenous factors and anthropometric characteristics [15-17].

The objective of this study is to determine the effect of the micronutrients supplementation and regular aerobic exercise during pregnancy on maternal endothelial function and neonatal oxidative stress. Furthermore, the effect of both interventions on the anthropometric parameters of the newborn will be evaluated. We expect that the results of this study will provide valuable information to design efficient public health policies during pregnancy aimed to prevent NTCD.

### Hypothesis

Supplementation with micronutrients or regular aerobic exercise improves endothelial function in the mother and reduces oxidative stress in newborn.

## Methods/Design

### Study Design

This is a single blind, randomized controlled 2 × 2 factorial trial designed to evaluate the effectiveness of micronutrients supplementation and regular aerobic exercise during pregnancy. This design provides the advantage of assessing the independent and cumulative effects of the interventions in nulliparous pregnant women.

### Interventions

All women included in the study will receive usual prenatal care according to WHO guidelines. The following intervention groups will be compared:

#### 1. Control Group

This group will receive the usual prenatal care according to both WHO and Colombian guidelines and placebo (maltodextrine). In Colombia, pregnant women receive ferrous sulphate, calcium, and folic acid during prenatal care. In addition, they receive counselling about breastfeeding, key signs and symptoms, diet and screening for pregnancy diseases. The Colombian guidelines do not include physical exercise prescription during pregnancy neither counselling from a physical therapist [18].

#### 2. Exercise Group

Regular aerobic exercise: walking (10 minutes), aerobic exercise (30 minutes), stretching (10 minutes), and relaxation exercises (10 minutes) during a three-month period. Exercise will be performed during 3 sessions per week. A physical therapist and a physical educator will supervise all sessions. The exercise-program is according to the American College of Obstetrics and Gynecology (ACOG) [18] and the American College of Sports Medicine (ACSM) exercise prescription [19]. Aerobic activities will be performed at moderate intensity (60-70% of maximal heart rate) measured by the 6-20 Borg's rating scale (PER). Each session will start with a 5 min warm up, followed by 30 min of aerobic activity, including a 5 min cool down. Subsequently, a 15 min strength training for upper limbs, lower limbs, and deep abdominal muscle stabilization. The last 5 min consist of stretching and relaxation exercises. (Table 1)

**Table 1:** Pregnancy 2 X 2 Factorial Design

	Regular Physical Exercise	
	
	-	+
**Micronutrients**		
-	Usual Prenatal Care + Placebo	Usual Prenatal Care + Regular physical exercise
+	Usual Prenatal Care + Micronutrients Supplementation	Usual Prenatal Care + Regular physical exercise + Micronutrients Supplementation

#### 3. Micronutrients Supplemental Group

This group will receive supplementation of zinc (30 mg), selenium (70 μg), vitamin A (400 μg), alphatocopherol (30 mg), vitamin C (200 mg), and niacin (100 mg).

#### 4. Combined interventions Group

This group will receive both the supplementation with micronutrients and physical exercise as described above.

### Sample size

Measurement of endothelial function (FMD), validated in several population studies was selected as the critical variable to calculate the sample size. Sample size was conservatively calculated to detect all six pairwise difference between treatment groups with a Bonferroni adjustment for an overall 0.05 type I error (0.05/6 = 0.0083) [20]. A sample size of 320 subjects or 80 per treatment group was estimated. The sample size was calculated assuming a specific difference between the groups of 4.3% in the FMD after 3 months of treatment with 11 of maximum standard deviation. It was accepted a type I error of 0.05, a power of 80%, and it was adjusted by 20% [20].

Eligible women for the present study and those interested in participating will be invited to a pre-test that includes an interview (Visit A) and further assessments that will be performed at the Red de Salud Ladera (Hospital Cañaveralejo, Centro de Salud Meléndez, Centro de Salud Siloé, Hospital Universitario del Valle) and at the Laboratory of Biochemistry of the Universidad of Valle, Cali-Colombia. The first visit will take place between weeks 12 and 20 (Visit B), the second at week 32 and 36 (Visit C) and the last one, after delivery (Visit D).

### Settings

The trial will be conducted in Cali, the third largest city of Colombia. The study will be carried out in six public health care institutions, which offer prenatal care in the first level of complexity to people living in the lowest socioeconomic stratum of the population. These institutions are located in the southwest area from Cali (*Red de Salud Ladera*) and provide services mainly for the government-subsidized population in the Colombian healthcare system. The Ethics Committees of Universidad del Valle and Red de Salud Ladera (Resolution-017/08-UV and SCAH/0408-A/08) approved the trial. All participants will provide written informed consent before entering the study.

### Outcome variables

1. The primary outcome is the change of endothelium-dependent brachial artery flow-mediated dilatation (FMD) after three months of regular aerobic exercise. [Time Frame: Baseline and 32 to 36 weeks of gestation].

2. The secondary outcome is the concentration of F2-isoprostanes in umbilical cord blood of newborn [Time Frame: Delivery].

### Analytic plan

All pregnant women studied will be analyzed according to original group allocation and all changes in outcome variables will be attributed to the assigned treatment. An exploratory analysis will be performed to determine frequency, range, variability, and distribution type for each variable in order to use the most appropriated statistical test when comparisons will be necessary. Subsequently, a descriptive analysis of the socio-demographic characteristics, gestational variables, and baseline measures (x_0_) will be performed for each comparison group. These analyses permit to assess the primary analysis of the data and will be undertaken using the principle of intention-to-treat (ITT). The ITT analysis for this study will include all participants, including those who are not fully compliant and those with missing outcome data. While we plan to implement procedures to minimize loss to follow-up and patient withdrawal, we expect to observe some attrition. We plan to employ multiple imputations to handle missing data in the analysis. Multiple imputations, compared to other case deletion strategies, can provide valid inferences with less restrictive assumptions surrounding the mechanism for missing data [21-23].

Due to that this is an experimental design with two measures of the maternal outcome variable, the first at baseline x_0 _(t_0 _= 16-20 gestational weeks) and the second after interventions x_1 _(t_1 _= 32-36 gestational weeks) in four study groups, a comparative analysis between these measures to establish differences will be executed. In order to perform these comparisons one way ANOVA [24] or Kruskal-Wallis [25] tests will be applied, if appropriated. Subsequently, a multivariate analysis will be carried out and the autocorrelation between repeated measures will be taking in to account. We will use longitudinal analysis methods such as GEE in order to control the differences among measures at baseline and to incorporate incomplete observations into this analysis [26].

In order to evaluate the effects of maternal regular physical exercise and micronutrients supplementation on newborn's oxidative stress (concentration of F_2_-Isoprostanes in umbilical cord blood), a comparative analysis will be executed to establish if the effects are different among study groups using analysis of covariance (ANCOVA), a form of general linear model [26]. We will include the baseline measure as a covariate in the model and pre-specified potential confounding factors: maternal age, previous physical exercise level, tobacco or alcohol consumption, differences in diet report and pregnancy complications such as hypertension and diabetes [26]. These will be included regardless of whether baseline imbalance exists. This approach has been chosen because confounder selection strategies that are based on collected data can result in models with poor statistical properties [26]. This will adjust appropriately for any baseline imbalance and will provide the most powerful analysis. Adjusted estimates of intervention effect from this model will be reported as the primary analysis in the trial publication.

Finally, we will investigate if there is an interaction between the two interventions for the primary outcome at 32 gestational weeks (regular physical exercise) or until the birth (micronutrients supplementation). For these analyses we will include appropriate interaction terms in the models [26]. The trial is not powered to detect these interactions and is likely to have poor precision for the interaction terms. We plan to report regression coefficients for the interaction terms and their 95% confidence intervals.

### Eligibility

Pregnant nulliparous women (n = 320) in a range of age between 16 to 30 years will be invited to participate in this randomized clinical trial. These women will not any indications of additional specialized care.

### Exclusion criteria

Pregnancy with history of high blood pressure, chronic medical illnesses (cancer, renal, endocrinologic, psychiatric, neurologic, infectious and cardiovascular diseases), persistent bleeding after week 12 of gestation, poorly controlled thyroid disease, placenta praevia, incompetent cervix, polyhydramnios, oligohydramnios, history of miscarriage in the last twelve months, diseases that could interfere with participation in the study (following recommendations from ACSM 2000, ACOG 2002) [18,19] or any medical condition are excluded from the trial.

### Inclusion Criteria

Nulliparous women not involved in a structured exercise program during pregnancy will be chosen, having a live fetus at the routine ultrasound scan, and a normal pregnancy, with gestational age 16 to 20 weeks. Written informed consent will be obtained from each woman prior to the inclusion in the study.

### Blinding and Randomization methods

**Figure 1:**
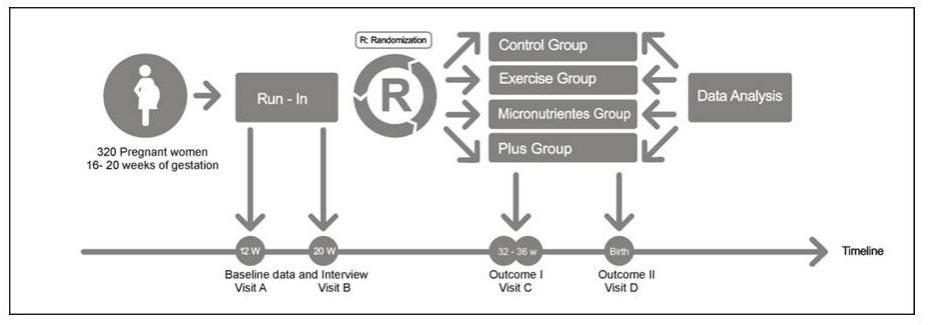
Study design

The randomization procedure into the two study arms was performed by the Fundación FES Social, Division de Salud, Cali-Colombia (Biometry and Epidemiology Department). The biometricians compiled an allocation list which was the basis for the pharmacists to prepare sequentially numbered envelopes containing two boxes of study medication for trial use. The boxes contained a zinc (30 mg), selenium (70 μg), vitamin A (400 μg), alphatocopherol (30 mg), vitamin C (200 mg), and niacin (100 mg) or an equivalent placebo capsule (maltodextrine). After inclusion into the trial, the study researcher assigned the content of the lowest numbered envelope to the participant. The principal investigator and coordinator the allocation sequence, and randomization is performed through a secure website which ensures allocation concealment. All participants and study personnel (including investigators, research coordinators, data analysts) will be blinded to treatment allocation throughout the trial (Table 1). Access to the allocation code will be restricted to one study statistician who will not perform the final study analyses. Also, neither the participant nor the study researcher knew about the content of the boxes, the capsule was not distinguishable. Since no specific qualifications or experience are required for the research assistants, training will be provided prior to the initiation of the trial about protocol and measurement procedures and methods used to maintain the blindness. These procedures are also detailed in the study operations manual. Moreover, the importance of maintaining the blinding and allocation concealment will be reinforced by regularly scheduled conference calls at the sites and daily meetings with the field investigators, Figure 1.

## Procedures

### Enrolment

A 15-month period is expected for enrolment of study participants. Screening of participants will consist in the evaluation of inclusion and exclusion criteria, explanation of the study protocol, and the assessment of the willingness to participate in the study, (Figure 1). Eligible subjects will be scheduled to the following visits:

**- Visit A**: consists of a structured validated interview (socio-demographic data, habits, and medical record) and a detailed physical examination by a nurse and a gynecologist. Prenatal care visits will be continued in the primary care center assigned to the healthcare insurance plan of each woman.

### Baseline assessments and active follow-up

**- Visit B**: measurements of blood pressure, anthropometric parameters, FMD, functional capacity (VO_2Max_), and electrocardiogram. During this visit, the participants will be randomized to one of the four intervention groups. For this purpose, a randomization system using maximum blocks of 10 will be used.

**- Visit C**: within 32 and 36 week period of gestation, measurements described above are repeated.

**- Visit D**: the following pregnancy outcomes will be recorded at postpartum: pregnancy duration, blood pressure, characteristics of delivery (route and duration of delivery, and postpartum haemorrhage), newborn outcomes (gestational age at birth, Apgar score, weight, height, head circumference, and abdominal circumference). With these measures, the following variables will be calculated: weight/height^3^, head circumference/birth weight, and weight/height [27]. In umbilical cord blood, measurements of the following outcomes will be obtained: oxidative stress biomarker (F_2_-isoprostanes). In addition, the number of prenatal controls and information regarding pregnancy complications will be registered.

### Passive follow-up

All the women included in the study will undergo a passive follow-up interview at day 30^th ^after delivery. This follow-up will be performed via phone or direct house visit in those cases where no phone number is available for contact.

### Physical measurements

Anthropometric parameters will be determined in each participant at enrolment and before delivery. All measurements will be performed in fasting patients, wearing light clothes and without shoes, using standardized formats and methods. In addition, these parameters will be taken in duplicate by the same examiner on each woman.

Weight will be measured with the patient in standing position and then registered with 200 g approximation. The weight scale will be calibrated to 0 before each measurement.

Height will be measured using a metric tape with the patient in standing and vertical position leaned against the wall in Frankfort's position, and the value marked by a ruler placed horizontally on the head of the patient.

Heart rate or number of beats per minute will be measured in the radial artery.

Blood pressure will be taken twice (with a 5 min period between the measurements) using a mercury sphyngomanometer on the right arm, with the patient comfortably seated, after a 5 min rest. Systolic blood pressure (SBP) will be determined by the first audible sound (Korotkoff phase 1). Diastolic blood pressure (DBP) will be registered when the sound disappears (Korotkoff phase 5) [28]. The patient should not have smoked 30 min prior to the blood pressure measurement. The pneumatic arm cuff must cover 2/3 of the upper arm's length; its inferior border must be 2-3 cm over the antecubital space; the cuff will be slowly deflated. The mean blood pressure (MBP), will be calculated using the following formula [SBP+(2 × DBP)]/3.

Body composition that includes indirect fat mass percentage (FM; kg) and fat-free soft tissue mass (FFT; kg) will be measured at baseline and follow-up visits using indirect formulas [29].

Body Mass Index (BMI) will be estimated using the weight in kg divided by the second power of the height expressed in meters.

Functional Capacity (VO_2max_) will be determined in order to find the pregnancy-specific anaerobic threshold (AT). All women will perform an incremental sub-maxim exercise test of the ramp type. This test will be carried out on a mechanically braked cycloergometer (Monarck 820K) at a bench height to facilitate the most effective pedalling. Participants will be instructed to perform the test for as long as possible to ensure a true maximal attempt. Standard ACSM [19] test termination criteria will be monitored and followed throughout each test. After a 3-min warm-up at 20 watts, the work rate will increase every minute by 15 watts. Throughout the test, the pedalling rate will be set up at 60 rpm. Blood pressure, heart rate and perception of exhaustion will be monitored every minute. The incremental workload will be discontinued when either heart rate of 160 beat per min or the maximum level of exhaustion on Borg's scale are reached; another 3-min workload will be performed at 10 watts followed for cooling down before completion of the exercise test.

### Measurement of flow-mediated dilatation of the brachial artery

Endothelium-dependent vasodilatation will be measured with the technique introduced by Silva *et al. *[30], using the guidelines reported by Corretti *et al. *[31] The diameter of the brachial artery will be assessed using a high-resolution ultrasound device (Siemens SG-60, USA), equipped with a 7.5 MHz linear array transducer and an integrated electrocardiography package. The ultrasound procedures will be performed with the subject resting quietly in supine position for at least 10 min. All measurements will be taken at the end of diastole observed by electrocardiogram. First, the diameter of the right brachial artery will be searched in a cross-sectional view and then scanned over a longitudinal section 5 to 10 cm proximal to the right elbow. The diameter of the brachial artery will be measured from the anterior to the posterior intima-lumen interface at a fixed distance. The mean diameter will be calculated from 4 cardiac cycles. After, a pneumatic tourniquet placed around the right forearm will be rapidly inflated to at least 50 mm Hg above the systolic blood pressure for 5 min. A sudden release of the cuff will induce an increase in blood flow in the brachial artery located proximal to the tourniquet. During reactive hyperemia, there will be an increase in shear stress, causing endothelium-dependent vasodilatation, mainly due to endothelial release of nitric oxide [32]. This secondary dilation enhances and prolongs the reactive hyperaemic phase. FMD of the brachial artery will be measured 45-60 s after cuff release. The change in diameter caused by the increased flow will be calculated as the percentage change relative to the baseline measurement (FMD%). The dilating brachial artery response due to shear stress has been shown to have good accuracy and reproducibility [30,31]. Images will be recorded on DVD player, for subsequent measurements by two observers blinded to the study, finally, both measurements will be averaged.

### Biochemical markers

The routine clinical test and those of the endothelial function and inflammation markers will be processed in the Proteins, Enzymes and Biochemistry Laboratory- Universidad of Valle (Cali, Colombia). The oxidative stress biomarker (F_2_-isoprostanes) will be measured in umbilical cord blood using gas chromatography-mass spectrometry (GC-MS) as previously described [33,34].

### Physical activity and health-related physical fitness

Physical activity will be assessed applying the adapted Spanish version from the Pregnancy Physical Activity Questionnaire (PPAQ) [35]. The PPAQ measures 1 to 5 physical activity levels. Moreover, other questionnaires will be administered to assess patterns and determinants related to physical activity such as sedentary behaviour, physical activity stages, family influence, strategies for behavioural changes, and environmental factors. In addition, quality of life questionnaire (SF Community - short-form survey (*SF*-*12*™) (^©^1998, 1999 QualityMetric Inc., Lincoln, RI) will be assessed to record details of current medications, smoking and alcohol use and dietary patterns [36].

### Nutrition

The pregnant women will answer three nutritional questions: (i) did the participant eat meat the week before, and if so, how much?; (ii) did the participant eat fried food the week before, and if so, how often?; and (iii) how often did the participant eat vegetables and carbohydrates during the previous week [37].

## Study conduct and monitoring

The study will be conducted according to Good Clinical Practice and to standard operating procedures. The Human Ethical Committee at the Universidad del Valle will monitor this clinical trial. The coordinating center will be composed by physical educators, physicians, gynaecologists, nurses, physical therapists, clinical epidemiologists, and bacteriologists. Preliminary monitoring reports will be submitted to the experts, focusing on patient intake, adherence to protocol, baseline comparability of treatment groups, completeness of data retrieval, and adverse events. All adverse events will also be reported to the Ethics Committee of the Universidad del Valle. To standardize study procedures, an operations manual has been written and comprehensive training sessions will be held prior to the initiation of the trial. In addition, there will be weekly conference calls between the sites, the coordinating center, and the research chief in order to review procedures and address problems. To ensure the uniformity and quality of the interventions at each site, experts in exercise physiology will train all the therapists prior to the trial initiation and consult with them on an ongoing basis. The coordinating center will supervise the procedures to ensure the quality and integrity of the data. These processes that include inspections of the data forms as they are received from locations and periodic reviews of the computer data files will be performed in order to identify out of range values and missing data forms.

### Ethical aspects

The clinical trial will be conducted according to the Helsinki's Declaration, the Good Clinical Practice Guidelines and the Colombian legislation (Resolution 8430/93 of the Ministry of Health). Each participant will provide a written informed consent in a form designed for such purpose. The information generated by the study will be confidential and strictly limited to the purposes stipulated in the protocol. The patient may refuse to continue participating in the study at any moment after providing consent. The study has been approved by both the Universidad of Valle and the Red de Salud Ladera Ethics committees. All assessments will be performed by trained staff. The blood samples will be collected in aseptic conditions by an expert bacteriologist. Design and methods of this RCT are in accordance with the recently published CONSORT guidelines for trials of nonpharmacological interventions (http://www.consort-statement.org) [38].

## Discussion

At the moment, most of efforts to prevent non-transmissible chronic diseases at population level have been centered in promoting healthy behaviors like physical activity, ingestion of fruits and vegetables, and promoting the reduction of tobacco and alcohol consumption in the adult population. Nonetheless, these public health policies to reduce the burden of non-transmissible chronic diseases have been largely inefficient. In the last years, many studies have indicated the relation between metabolic alterations and fetal growth with the development of non-transmissible chronic diseases in the adulthood. More recently, it has been proposed that maternal and placental factors such as endothelial function and oxidative stress are the precursory mechanisms of fetal metabolic alterations and the posterior development of non-transmissible chronic diseases. Also, it has been suggested that supplementation with micronutrients and regular physical exercise [39,40] during the gestation can regulate these maternal and placental factors. For the reasons just mentioned, it is necessary to clarify if oxidative stress and endothelial dysfunction are related to fetal metabolic alterations and if the supplementation during the gestation with micronutrients and/or the regular physical exercise can regulate them, which would be an early and novel alternative to fortify the prevention of non-transmissible chronic diseases in the population.

### Study timeline

**Figure 2:**
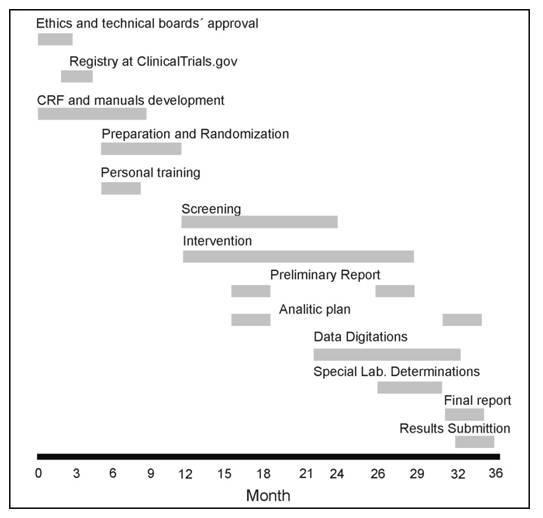
**Study Timeline**. CRF: Case report format.

The study will last 36 months. Figure 2.

## Abbreviations

ACOG: American College of Obstetrics and Gynecology; ACSM: American College of Sports Medicine; BMI: Body mass index; CRF: Case report format; DBP: Diastolic blood pressure; FMD: Flow mediated dilatation; PPAQ: Pregnancy Physical Activity Questionnaire; MBP: Mean blood pressure; PER: Borg's rating scale; PC: Prenatal care; SBP: Systolic blood pressure; SF-12: The SF Community - short-form survey; W: Wattios

## Competing interests

The authors declare that they have no competing interests.

## Authors' contributions

RRV, JCM, ACA, IE, JGO contributed in the conception and design of the manuscript. He also revised it critically and gave the final approval of the version published. MR, MM, SLG, BS and WS also participated in the conception and design of the manuscript, additionally they revised it critically.
